# Review: Emerging concepts in the pathogenesis of tendinopathy

**DOI:** 10.1016/j.surge.2017.05.005

**Published:** 2017-12

**Authors:** Benjamin J.F. Dean, Stephanie G. Dakin, Neal L. Millar, Andrew J. Carr

**Affiliations:** a Nuffield Department of Orthopaedics, Rheumatology and Musculoskeletal Sciences (NDORMS), University of Oxford, Botnar Research Centre, Windmill Road, Oxford, OX3 7LD, UK; b Institute of Infection, Immunity and Inflammation, College of Medicine, Veterinary and Life Sciences University of Glasgow, Glasgow, Scotland, UK

**Keywords:** Tendon, Tendinopathy, Tendinitis, Inflammation, Pain, Pathogenesis

## Abstract

Tendinopathy is a common clinical problem and has a significant disease burden attached, not only in terms of health care costs, but also for patients directly in terms of time off work and impact upon quality of life. Controversy surrounds the pathogenesis of tendinopathy, however the recent systematic analysis of the evidence has demonstrated that many of the claims of an absence of inflammation in tendinopathy were more based around belief than robust scientific data. This review is a summary of the emerging research in this topical area, with a particular focus on the role of neuronal regulation and inflammation in tendinopathy.

## Introduction

Tendinopathy is a common clinical problem, the three most common sites affected are the Achilles, patellar and rotator cuff tendons.^1^ The rotator cuff tendons are the most commonly affected with an annual incidence of over 1% that increases with age^2^, ^4^ and consequently there is a rising rate of surgery for rotator cuff tears.^5^ Others include the tendons around the elbow (golfer's and tennis elbow) and the tendons around the wrist. There is a significant disease burden attached to painful tendinopathy, not only in terms of health care costs, but also for patients directly in terms of time off work and impact upon quality of life. The primary purpose of this review is not to give an overall summary relating to all tendinopathies, it is to summarise specific emerging areas relating to tendinopathy pathogenesis research in which the authors have a particular expertise while giving a brief overall context this recent research.

## Aetiology and pathogenesis

The pathogenesis of tendinopathy is certainly multifactorial and complex.^6^, ^7^ Increased age is a key risk factor for the development of tendinopathy,^8^, ^9^ although the commonly affected tendons all experience high levels of mechanical stress^10^ and over-use is a frequently implicated risk factor.^8^, ^11^ The mechanism of overuse has been well demonstrated in animal models,^12^, ^13^ while both metabolic and vascular risk factors are associated with the development of tendinopathy.^9^, ^14^ Inactivity and the unloading also have an effect on tendon collagen homeostasis.^15^

Recent systematic reviews have clearly demonstrated that patients with high cholesterol and diabetes are at significantly higher risk of developing tendinopathy,^14^, ^16^ while recent review has demonstrated that an association exists between the metabolic-hormonal imbalances and tendon degeneration.^17^ Hypercholesterolaemia has also been demonstrated to have a significant impact upon tendon repair *in vivo*,^18^ while the clear link between hypercholesterolaemia and inflammation has been long known.^19^ This emerging link between metabolic dysregulation and chronic inflammation in tendinopathy has also been supported by a recent study using Achilles tendon biopsies from a group of patients.^20^

The historical context relating to the rotator cuff provides an interesting insight into the frequent debates and changing viewpoints as regards tendinopathy pathogenesis. Codman initially proposed in 1934^21^ that degeneration within the tendon was the ‘intrinsic’ primary causal factor. The ‘extrinsic’ theory relating to tendon damage secondary to attrition by surrounding structures was popularised by Neer^22^ and the term ‘impingement’ was coined. Broadly the modern consensus recognises both the role of intrinsic and extrinsic factors, but sees the intrinsic factors as being more dominant overall^23^ with the use of the term ‘impingement’ appearing increasingly baseless.^24^ It is probable that different patients have different disease phenotypes with different intrinsic and extrinsic factors playing variable roles. Certainly not all tendinopathies are identical, as represented by differences in both the tendon's local anatomy and epidemiological profile.

## Histopathology and clinical features

Tendinopathy has characteristic histopathological, clinical and radiological findings.^25^ The histopathological changes include collagen disorganisation, the increased deposition of mucoid ground substance, increased overall cellularity, as well as the appearance of round and plump ‘chondroid’ type cells.^26^, ^27^ These features of apparent attempted healing diminish as degree of tendon degeneration increase. The overall picture is one of pathological chondroplasia in which tissue which normally exhibits a tensional morphology is replaced by tissue of a fibrocartilage-like phenotype.^26^, ^28^ Historically several different words have been used to described tendon related pathology including ‘tendinosis’ (implying degenerative aetiology), ‘tendinitis’ (implying inflammatory aetiology) and the more recently favoured and less aetiologically specific ‘tendinopathy’.^29^ This diversity of language reflects a historical disagreement within the scientific community as to the exact role of inflammation in the aetiology of ‘tendinopathy’. Recent evidence has shown that tendon overload is linked to alterations in cell shape, as well as increased markers of inflammation and matrix degradation.^30^ The way in which the cells interact with the extracellular matrix is an area of much interest^31^, ^32^; inflammation and damage-induced matrix remodelling seem to be concentrated in, or in the vicinity of, the highly cellular interfascicular matrix.^33^ It may be therefore be postulated that interactions between the tendon, the interfascicular matrix and adjacent fat pads are instrumental in the development of tendinopathy, with the latter being a key potential source of key cytokines and inflammatory cells.^34^ This may help explain the presence of persistent inflammation in tendinopathy,^35^ a phenomenon which has been shown to have important effects on tendon cells *in vitro*.^36^, ^37^

Clinical symptoms including pain are frequently poorly matched to the histopathological and radiological findings, meaning that a high proportion of patients with a tendon that is both histopathologically and radiologically degenerate experience have no related pain or symptoms.^38^ The reasons for this mismatch between pathology and perceived pain are poorly understood, however recent research has identified the peripheral and central pain processing pathways as good candidates for an explanation.^39^, ^40^ It appears that the presence of pain in tendinopathy not only relates to mechanical changes in the tendon but also changes to the ways in which the local cells and the peripheral nerves react to this change, thus contributing to the nociceptive pathways to higher centres being activated. Overall the vast majority of tendon ruptures (97%) occur in patients with histopathologically abnormal tendons.^41^

## Neuronal pathways and glutamate

The neuronal response to tendon injury involves nerve in growth during the initial inflammatory phase; the subsequent proliferative and remodelling phases are regulated by sensory nerves, as well as the glutaminergic and autonomic systems.^42^ Glutamate is an important amino acid involved in many key physiological processes including cell metabolism, pain sensitization and collagen synthesis.^39^, ^43^ Glutamate receptors can be broadly broken down into two major types: inotropic, which are glutamate-gated ion channels (iGlu), and metabotropic, which are G-protein coupled receptors that modulate signal transduction cascades (mGlu).^44^ The inotropic receptors include Kainate (KA) receptors, α-amino-3-hydroxy-5-methyl-4-isoxazole propionic acid (AMPA) receptors and N-Methyl-d-Aspartate (NMDA) receptors.^45^ Glutamate has been shown to induce pain and hyperalgesia when injected around human tendon tissue.^46^ An upregulation of the glutaminergic signalling has been linked to inflammatory change in a rat supraspinatus model.^47^

The first study to recognize the presence of glutamate in tendinopathy used a microdialysis technique in chronic painful Achilles tendinopathy.^48^ Glutaminergic changes have since become increasingly described in painful tendinopathy,^42^ including an increase in extracellular glutamate concentration and the up-regulation of N-Methyl-d-Aspartate (NMDA) receptors.^49^, ^50^ Recently the upregulation of the glutaminergic system has been confirmed to be present in rotator cuff tendinopathy for the first time.^51^ This histological and immunohistochemical study demonstrated that an increase in glutamate staining was present in the painful tendinopathic rotator cuff tendons alongside the classical histological changes which included increased collagen disorganization and cellularity. Glutamate staining was distinctly expressed in resident cells within the tendon. The release of glutamate from tendon cells was first hypothesized by Scott et al.^52^ who detected vesicular glutamate transporter expression in cells localized in tendon tissue in lower limb tendinopathies. This was later detected by Schizas et al.^50^ Because of the mechanical and structural differences between tendon locations, it cannot be assumed that cell behaviour is uniform in both upper and lower limb tendinopathies. This study has also shown significant staining of certain ionotropic and metabotropic receptors on tendon cells residing in damaged rotator cuff tissue, for example NMDAR1 and mGluR1. This study did not find any correlation with the severity of patient pain symptoms. This is not unsurprising as previous studies have failed to detect this link.^53^ Previous studies of tendon structure has been shown to be a powerful predictor of the presence of pain,^38^ but not of its severity.

Sensory neuropeptide expression has also been shown to be associated with both failed healing and pain in an animal model of tendon injury,^54^ they have also been causally linked with tendinopathy-like changes in animal models.^55^, ^56^ Neuropeptides have pro-proliferative, angiogenic and stem cell-stimulating properties *in vitro*,^57^ however little work had been carried out to investigate the effects of glutamate on tendon derived cells. Dean et al. exposed tendon derived cells from both healthy controls and patients with tendinopathy to different concentrations of glutamate and specific glutamate receptor inhibitors.^58^ Tendon derived cell viability was reduced after 72 h of exposure to relatively low concentrations of glutamate (0.05 mM and 1.875 mM) and this deleterious effect was attenuated by NMDAR antagonism. Higher concentrations of glutamate reduced cell viability at 24 h in tendon tear derived cells and not in control cells. A reduction in collagen (COL1A1 and COL3A1) and increase in aggrecan gene expression were seen after both 24 and 72 h of glutamate exposure. Overall the *in vitro* effects of glutamate in terms of reducing cell viability, decreasing collagen gene expression and increasing aggrecan gene expression suggested that the raised levels of glutamate contributes to the pathogenesis of tendinopathy.

## Clinical meaning of neuronal changes

Tendon samples from patients with persistent pain demonstrated increased levels of metabotropic glutamate receptor 2 (mGluR2), Kainate receptor 1 (KA1), Protein Gene Product 9.5 (PGP9.5), CD206 (macrophage marker) and CD45 (pan-leucocyte marker) versus pain-free controls.^59^ Notably the painful and pain-free patient groups were matched in terms for basic demographic and tendon structure, while there were no differences between groups in terms of the basic histology. NMDAR1 co-localised with CD206 positive cells, whereas PGP9.5 and glutamate were predominantly expressed by resident tendon cells. Within the gene expression data related to cells derived from rotator cuff years there were strong correlations between CD206 expression and glutamate receptor expression. This work demonstrated an association between glutaminergic and pro-inflammatory changes in tendon cells and pain. To our knowledge this is the first histological study that has used structurally and age matched tendon from pain-free tendinopathic patients as a control. The co-localisation of NMDAR1 and CD206 suggests that certain glutamate receptors are predominantly expressed on ‘inflammatory’ type cells within tendon.

Recent *in vivo* work by Valkering et al. has investigated the role of glutamate in Achilles tendon healing following acute Achilles tendon rupture.^60^ Patients were randomised into two groups, and those in the functional weight bearing group had significantly higher levels of glutamate than the non-weight bearing group, while the higher glutamate levels correlated with both the level of the marker of procollagen type I (PINP) and improved functional outcome at six months. Substance P enhances cell proliferation and sensory nerve in growth in the rat,^61^ conversely joint immobilisation has been shown to reduce the expression of sensory neuropeptides in the rat and this is associated with significantly poorer tendon healing.^62^ In clinical terms glutamate appears important in normal tendon healing,^63^ while its upregulation in tendinopathy appears consistent with an environment of persistently failing healing and perhaps a failure of inflammation to resolve.

## The emergence of inflammation

The dwindling of popularity of the term ‘tendinitis’ represented skepticism regarding the role of inflammation in tendon degeneration. Recent systematic analysis of the evidence has demonstrated that many of the claims of an absence of inflammation in tendinopathy were more based around belief than robust scientific data.^29^ Several studies have stated that ‘no inflammatory cells’ were present in samples from tendinopathic patients but had only looked for neutrophils, not other types of ‘inflammatory cell’. In fact the evidence to support the role of inflammation in tendinopathy pathogenesis has become increasingly overwhelming in recent years with a majority of studies demonstrating increased numbers of macrophages in diseased tendons.^29^

Macrophages are known to play an essential role orchestrating inflammation and tissue repair. The signalling pathways underpinning activation of macrophages to become M1 or M2 subtypes have been revised to identify the signalling pathways underpinning macrophage activation interferon, NF-κB, (STAT6) and glucocorticoid receptor activation pathways.^64^ These macrophage activation pathways have recently been identified in samples of diseased human rotator cuff tendons. Tendon tissues from patients with early stage disease showed increased expression of genes and proteins induced by Interferons and NF-κB^37^. Conversely, tendons from patients with advanced stage disease (large to massive tears) showed expression of genes and proteins induced by STAT6 and glucocorticoid receptor activation pathways. These findings highlight the complexity of inflammatory processes in diseased tendons and how they might change with the stage of disease.

Increasing evidence has shown that inflammatory mechanisms and the innate immune system are activated within the tendon matrix microenvironment during tissue injury and dysregulated homeostasis. An essential component of the regulatory process that drives tendon remodelling includes cytokines that dictate cell type and tissue specificity of responses that ultimately balance a reparative versus degenerative process. Endogenous expression of TNFα, IL-1β, IL-6, IL-10, VEGF and TGFβ has been demonstrated in tenocytes^65^, ^66^, ^67^, ^68^ while diseased human tendon has shown expression of Such expression is functionally implicated *in vivo*: for example, the mechanical properties of healing tendons in IL-6^−/−^ mice were inferior compared with littermate controls.^69^

Recent mechanistic dissection has highlighted a role for the cytokine IL-33, a member of the IL-1 cytokine family that plays a major role in innate and acquired immune responses, in matrix/inflammatory crosstalk in tendon damage.^70^ IL-33 message and protein expression was significantly increased in early human tendinopathy compared to both established tendinopathy and normal tendon while the addition of exogenous IL-33 to *in vitro* human tenocyte cultures resulted in increased expression of type I but particularly type III collagen mRNA/protein. Moreover, an *in vivo* patellar tendon injury model the addition of IL-33 significantly reduced the tendon strength (load to failure) of WT mice by ∼30% at early time points, likely as a consequence of the concomitant collagen 3 matrix changes, which result in mechanically inferior tendon. Taken together these studies demonstrate a key functional role for IL-33 in early injury induced matrix dysregulation and subsequent cytokine feedback mechanisms that have an ultimate biomechanical and clinical effect.

Emerging studies highlight microRNAs(miR) as key regulators of leukocyte function and the cytokine network while orchestrating proliferation and differentiation of stromal lineages that determine extracellular matrix composition.^71^ We identified reduced expression of mio-29a, which directly targets numerous extracellular matrix genes and is implicated in the regulation of innate and adaptive immunity, in human biopsies and demonstrated that its reduction leads to development of tendinopathy. In human tenocytes, miR-29a was only capable of influencing the expression of collagen type 3 and not type 1. This work has been expanded to an equine tendinopathy model,^72^ considered equivalent to human disease, reinforcing that injury-induced loss of miR-29a is responsible for the over-expression of col3 seen in equine tendinopathy. Collectively these data suggest that the reintroduction of *miR-29a* to the injury-induced miR-29a deficiency in tendon could reverse the key collagen switch that remains a core pathological feature of tendinopathy. Thus while the functional contribution of cytokine biology and its downstream consequences in tendinopathy remains to be established there is now a convincing scientific rationale towards translational immunobiology to benefit tendinopathy patients.^73^ The short term benefits of anti-inflammatory drug treatments in tendinopathy have been demonstrated in the short term,^74^ however there remains a distinct lack of effective long term treatments of any form.^75^

## Conclusions

Fundamentally a better understanding of the pathogenesis of tendinopathy and the underlying mechanisms is essential if we are to develop more effective long term treatment strategies for the management of tendinopathy.^76^, ^77^ Our improved understanding which includes this work relating to the emerging roles of the glutaminergic and inflammatory systems means that more effective novel treatments may be just around the corner.

## Sources of financial support

No specific funding was received relating to this review article.

BD received funding for his PhD from Orthopaedic Research UK (501) and the Jean Shanks Foundation. NM receives funding from the Wellcome Trust (WT100651MA), Royal College of Surgeons of Edinburgh and Arthritis Research UK. SGD is funded by Arthritis Research UK (grant 20506). AJC is supported by the National Institute for Health Research Biomedical Research Unit (HFR01921) and invention for innovation (i4i), MRC confidence in concepts and Wellcome Trust Health Innovation Challenge fund programmes.

## References

[1] Wilson J.J., Best T.M. (Sep 1 2005). Common overuse tendon problems: a review and recommendations for treatment. Am Fam Phys.

[2] Littlewood C., May S., Walters S. (2013). Epidemiology of rotator cuff tendinopathy: a systematic review. Shoulder Elb.

[4] Teunis T., Lubberts B., Reilly B.T., Ring D. (Dec 2014). A systematic review and pooled analysis of the prevalence of rotator cuff disease with increasing age. J Shoulder Elb Surgery/Am Shoulder Elb Surg.

[5] Paloneva J., Lepola V., Aarimaa V., Joukainen A., Ylinen J., Mattila V.M. (Aug 12 2015). Increasing incidence of rotator cuff repairs–A nationwide registry study in Finland. BMC Musculoskelet Disord.

[6] Xu Y., Murrell G.A. (Jul 2008). The basic science of tendinopathy. Clin Orthop Relat Res.

[7] Riley G. (Feb 2004). The pathogenesis of tendinopathy. A molecular perspective. Rheumatology (Oxford).

[8] de Jonge S., van den Berg C., de Vos R.J. (Oct 2011). Incidence of midportion Achilles tendinopathy in the general population. Br J Sports Med.

[9] Tashjian R.Z. (Oct 2012). Epidemiology, natural history, and indications for treatment of rotator cuff tears. Clin Sports Med.

[10] Neviaser A., Andarawis-Puri N., Flatow E. (Feb 2012). Basic mechanisms of tendon fatigue damage. J Shoulder Elb Surgery/Am Shoulder Elb Surg.

[11] Heir T., Glomsaker P. (Jun 1996). Epidemiology of musculoskeletal injuries among Norwegian conscripts undergoing basic military training. Scand J Med Sci Sports.

[12] Glazebrook M.A., Wright J.R., Langman M., Stanish W.D., Lee J.M. (Jun 2008). Histological analysis of achilles tendons in an overuse rat model. J Orthop Res Off Publ Ortho Res Soc.

[13] Soslowsky L.J., Thomopoulos S., Tun S. (Mar-Apr 2000). Neer Award 1999. Overuse activity injures the supraspinatus tendon in an animal model: a histologic and biomechanical study. J Shoulder Elb Surgery/Am Shoulder Elb Surg.

[14] Tilley B.J., Cook J.L., Docking S.I., Gaida J.E. (Dec 2015). Is higher serum cholesterol associated with altered tendon structure or tendon pain? A systematic review. Br J Sports Med.

[15] Dideriksen K., Boesen A.P., Reitelseder S. (Feb 01 2017). Tendon collagen synthesis declines with immobilization in elderly humans: no effect of anti-inflammatory medication. J Appl Physiol Bethesda Md 1985.

[16] Ranger T.A., Wong A.M., Cook J.L., Gaida J.E. (Aug 2016). Is there an association between tendinopathy and diabetes mellitus? A systematic review with meta-analysis. Br J Sports Med.

[17] Oliva F., Piccirilli E., Berardi A.C., Frizziero A., Tarantino U., Maffulli N. (Mar 2016). Hormones and tendinopathies: the current evidence. British Medical Bulletin.

[18] Hast M.W., Abboud J.A., Soslowsky L.J. (Jul 2014). Exploring the role of hypercholesterolemia in tendon health and repair. Muscles Ligaments Tendons J.

[19] Steinberg D. (Nov 2002). Atherogenesis in perspective: hypercholesterolemia and inflammation as partners in crime. Nat Med.

[20] Pingel J., Petersen M.C., Fredberg U. (2015). Inflammatory and metabolic alterations of Kager's fat pad in chronic achilles tendinopathy. PloS One.

[21] Codman E.A., Akerson I.B. (Jan 1931). The pathology associated with rupture of the supraspinatus tendon. Ann Surg.

[22] Neer C.S. (Jan 1972). Anterior acromioplasty for the chronic impingement syndrome in the shoulder: a preliminary report. J Bone Joint Surg Am.

[23] Lewis J.S. (Apr 2009). Rotator cuff tendinopathy. Br J Sports Med.

[24] Papadonikolakis A., McKenna M., Warme W., Martin B.I., Matsen F.A. (Oct 5 2011). Published evidence relevant to the diagnosis of impingement syndrome of the shoulder. J Bone Joint Surg Am.

[25] Riley G. (Feb 2008). Tendinopathy–from basic science to treatment. Nat Clin Pract Rheumatol.

[26] Dean B.J., Franklin S.L., Carr A.J. (Jul 2012). A systematic review of the histological and molecular changes in rotator cuff disease. Bone Joint Res.

[27] Khan K.M., Cook J.L., Bonar F., Harcourt P., Astrom M. (Jun 1999). Histopathology of common tendinopathies. Update and implications for clinical management. Sports Med Auckland NZ.

[28] Archambault J.M., Jelinsky S.A., Lake S.P., Hill A.A., Glaser D.L., Soslowsky L.J. (May 2007). Rat supraspinatus tendon expresses cartilage markers with overuse. J Orthop Res Off Publ Ortho Res Soc.

[29] Dean B.J., Gettings P., Dakin S.G., Carr A.J. (Feb 2016). Are inflammatory cells increased in painful human tendinopathy? A systematic review. Br J Sports Med.

[30] Thorpe C.T., Chaudhry S., Lei (Aug 2015). Tendon overload results in alterations in cell shape and increased markers of inflammation and matrix degradation. Scand J Med Sci Sports.

[31] Birch H.L., Thorpe C.T., Rumian A.P. (Jan 2013). Specialisation of extracellular matrix for function in tendons and ligaments. Muscles Ligaments Tendons J.

[32] Screen H.R., Berk D.E., Kadler K.E., Ramirez F., Young M.F. (Jun 2015). Tendon functional extracellular matrix. J Orthop Res Off Publ Ortho Res Soc.

[33] Spiesz E.M., Thorpe C.T., Chaudhry S. (Jun 2015). Tendon extracellular matrix damage, degradation and inflammation in response to in vitro overload exercise. J Orthop Res Off Publ Ortho Res Soc.

[34] Ward E.R., Andersson G., Backman L.J., Gaida J.E. (Dec 2016). Fat pads adjacent to tendinopathy: more than a coincidence?. Br J Sports Med.

[35] Dakin S.G., Buckley C.D., Al-Mossawi M.H. (Jan 25 2017). Persistent stromal fibroblast activation is present in chronic tendinopathy. Arthritis Res Ther.

[36] Al-Sadi O., Schulze-Tanzil G., Kohl B. (Jul 2011). Tenocytes, pro-inflammatory cytokines and leukocytes: a relationship?. Muscles Ligaments Tendons J.

[37] Dakin S.G., Martinez F.O., Yapp C. (2015). Inflammation activation and resolution in human tendon disease. Sci Transl Med.

[38] Yamaguchi K., DK, Middleton W.D., Hildebolt C.F., Galatz L.M., Teefey S.A. (2006). The demographic and morphological features of rotator cuff disease. A comparison of asymptomatic and symptomatic shoulders. JBJS (Am).

[39] Dean B.J., Gwilym S.E., Carr A.J. (Nov 2013). Why does my shoulder hurt? A review of the neuroanatomical and biochemical basis of shoulder pain. Br J Sports Med.

[40] Gwilym S.E., Oag H.C., Tracey I., Carr A.J. (Apr 2011). Evidence that central sensitisation is present in patients with shoulder impingement syndrome and influences the outcome after surgery. J Bone Joint Surg Br.

[41] Kannus P., Jozsa L. (Dec 1991). Histopathological changes preceding spontaneous rupture of a tendon. A controlled study of 891 patients. J Bone Joint Surg Am.

[42] Dean B.J., Franklin S.L., Carr A.J. (Apr 23 2013). The peripheral neuronal phenotype is important in the pathogenesis of painful human tendinopathy: a systematic review. Clin Orthop Relat Res.

[43] Newsholme P., Procopio J., Lima M.M., Pithon-Curi T.C., Curi R. (Mar 2003). Glutamine and glutamate–their central role in cell metabolism and function. Cell Biochem Funct.

[44] Julio-Pieper M., Flor P.J., Dinan T.G., Cryan J.F. (Mar 2011). Exciting times beyond the brain: metabotropic glutamate receptors in peripheral and non-neural tissues. Pharmacol Rev.

[45] Madden D.R. (Feb 2002). The structure and function of glutamate receptor ion channels. Nat Rev Neurosci.

[46] Gibson W., Arendt-Nielsen L., Sessle B.J., Graven-Nielsen T. (Apr 2009). Glutamate and capsaicin-induced pain, hyperalgesia and modulatory interactions in human tendon tissue. Exp Brain Res Exp Hirnforschung Exp Cereb.

[47] Molloy T.J., Kemp M.W., Wang Y., Murrell G.A. (Dec 2006). Microarray analysis of the tendinopathic rat supraspinatus tendon: glutamate signaling and its potential role in tendon degeneration. J Appl Physiol.

[48] Alfredson H., Thorsen K., Lorentzon R. (1999). In situ microdialysis in tendon tissue: high levels of glutamate, but not prostaglandin E2 in chronic Achilles tendon pain. Knee Surg Sports Traumatol Arthrosc.

[49] Alfredson H., Forsgren S., Thorsen K., Lorentzon R. (Sep 2001). In vivo microdialysis and immunohistochemical analyses of tendon tissue demonstrated high amounts of free glutamate and glutamate NMDAR1 receptors, but no signs of inflammation, in Jumper's knee. J Orthop Res Off Publ Ortho Res Soc.

[50] Schizas N., Lian O., Frihagen F., Engebretsen L., Bahr R., Ackermann P.W. (Apr 2010). Coexistence of up-regulated NMDA receptor 1 and glutamate on nerves, vessels and transformed tenocytes in tendinopathy. Scand J Med Sci Sports.

[51] Franklin S.L., Dean B.J., Wheway K., Watkins B., Javaid M.K., Carr A.J. (May 28 2014). Up-regulation of glutamate in painful human supraspinatus tendon tears. Am J Sports Med.

[52] Scott A., Alfredson H., Forsgren S. (May 2008). VGluT2 expression in painful Achilles and patellar tendinosis: evidence of local glutamate release by tenocytes. J Orthop Res Off Publ Ortho Res Soc.

[53] Dunn W.R., Kuhn J.E., Sanders R. (May 21 2014). Symptoms of pain do not correlate with rotator cuff tear severity: a cross-sectional study of 393 patients with a symptomatic atraumatic full-thickness rotator cuff tear. The Journal of bone and joint surgery. American Vol.

[54] Lui P.P., Chan L.S., Fu S.C., Chan K.M. (Apr 2010). Expression of sensory neuropeptides in tendon is associated with failed healing and activity-related tendon pain in collagenase-induced tendon injury. Am J Sports Med.

[55] Backman L.J., Andersson G., Wennstig G., Forsgren S., Danielson P. (Jun 2011). Endogenous substance P production in the Achilles tendon increases with loading in an in vivo model of tendinopathy-peptidergic elevation preceding tendinosis-like tissue changes. J Musculoskelet Neuronal Interact.

[56] Andersson G., Backman L.J., Scott A., Lorentzon R., Forsgren S., Danielson P. (Oct 2011). Substance P accelerates hypercellularity and angiogenesis in tendon tissue and enhances paratendinitis in response to Achilles tendon overuse in a tendinopathy model. Br J Sports Med.

[57] Backman L.J., Fong G., Andersson G., Scott A., Danielson P. (2011). Substance P is a mechanoresponsive, autocrine regulator of human tenocyte proliferation. PloS One.

[58] Dean B.J., Snelling S.J., Dakin S.G., Javaid M.K., Carr A.J. (Oct 2015). In vitro effects of glutamate and N-methyl-D-aspartate receptor (NMDAR) antagonism on human tendon derived cells. J Orthop Res Off Publ Ortho Res Soc.

[59] Dean B.J.F., SS, Dakin S., Murphy R., Javaid M.K., Carr A.J. (2015). Differences in glutamate receptors and inflammatory cell numbers are associated with the resolution of pain in human rotator cuff tendinopathy. Arthritis Res Ther.

[60] Valkering K.P., Aufwerber S., Ranuccio F. (Jun 2017). Functional weight-bearing mobilization after Achilles tendon rupture enhances early healing response: a single-blinded randomized controlled trial. Knee Surg Sports Traumatol Arthrosc.

[61] Carlsson O., Schizas N., Li J., Ackermann P.W. (Aug 2011). Substance P injections enhance tissue proliferation and regulate sensory nerve ingrowth in rat tendon repair. Scand J Med Sci Sports.

[62] Bring D.K., Reno C., Renstrom P., Salo P., Hart D.A., Ackermann P.W. (Feb 2009). Joint immobilization reduces the expression of sensory neuropeptide receptors and impairs healing after tendon rupture in a rat model. J Orthop Res Off Publ Ortho Res Soc.

[63] Greve K., Domeij-Arverud E., Labruto F. (Aug 2012). Metabolic activity in early tendon repair can be enhanced by intermittent pneumatic compression. Scand J Med Sci Sports.

[64] Murray P.J., Allen J.E., Biswas S.K. (Jul 17 2014). Macrophage activation and polarization: nomenclature and experimental guidelines. Immunity.

[65] Pufe T., Petersen W., Tillmann B., Mentlein R. (Oct 2001). The angiogenic peptide vascular endothelial growth factor is expressed in foetal and ruptured tendons. Virchows Arch.

[66] Tsuzaki M., Guyton G., Garrett W. (Mar 2003). IL-1 beta induces COX2, MMP-1, -3 and -13, ADAMTS-4, IL-1 beta and IL-6 in human tendon cells. J Orthop Res.

[67] Tohyama H., Yasuda K., Uchida H., Nishihira J. (Sep 2007). The responses of extrinsic fibroblasts infiltrating the devitalised patellar tendon to IL-1beta are different from those of normal tendon fibroblasts. J Bone Joint Surg Br.

[68] John T., Lodka D., Kohl B. (Aug 2010). Effect of pro-inflammatory and immunoregulatory cytokines on human tenocytes. J Orthop Res.

[69] Lin T.W., Cardenas L., Glaser D.L., Soslowsky L.J. (2006). Tendon healing in interleukin-4 and interleukin-6 knockout mice. J Biomech.

[70] Millar N.L., Gilchrist D.S., Akbar M. (Apr 10 2015). MicroRNA29a regulates IL-33-mediated tissue remodelling in tendon disease. Nat Commun.

[71] Brown B.D., Naldini L. (Aug 2009). Exploiting and antagonizing microRNA regulation for therapeutic and experimental applications. Nat Rev Genet.

[72] Millar N.L., Watts A.E., Akbar M., Hughes T., Kitson S., Gilchrist D.S. (2016). MicroRNA-29a in equine tendinopathy – a translational target. Equine Vet J.

[73] Millar N.L., Dean B.J., Dakin S.G. (Jun 3 2016). Inflammation and the continuum model: time to acknowledge the molecular era of tendinopathy. Br J Sports Med.

[74] Gaujoux-Viala C., Dougados M., Gossec L. (Dec 2009). Efficacy and safety of steroid injections for shoulder and elbow tendonitis: a meta-analysis of randomised controlled trials. Ann Rheum Dis.

[75] van Sterkenburg M.N., de Jonge M.C., Sierevelt I.N., van Dijk C.N. (Nov 2010). Less promising results with sclerosing ethoxysclerol injections for midportion achilles tendinopathy: a retrospective study. Am J Sports Med.

[76] Freedman B.R., Gordon J.A., Soslowsky L.J. (Apr 2014). The Achilles tendon: fundamental properties and mechanisms governing healing. Muscles Ligaments Tendons J.

[77] Millar N.L., Murrell G.A., McInnes I.B. (Jan 25 2017). Inflammatory mechanisms in tendinopathy – towards translation. Nat Rev Rheumatol.

